# Perceived Stigma and Associated Factors among People with Schizophrenia at Amanuel Mental Specialized Hospital, Addis Ababa, Ethiopia: A Cross-Sectional Institution Based Study

**DOI:** 10.1155/2014/694565

**Published:** 2014-05-21

**Authors:** Berhanu Boru Bifftu, Berihun Assefa Dachew

**Affiliations:** College of Medicine and Health Science, Department of Nursing, Gondar University, P.O. Box 196, Gondar, Ethiopia

## Abstract

*Background*. While effective treatments are available for people with schizophrenia, presence of perceived stigma prevents them from accessing and receiving the help they need to get. *Objectives*. To assess the prevalence and associated factors of perceived stigma among people with schizophrenia attending the Outpatient Department of Amanuel Mental Specialized Hospital, Addis Ababa, Ethiopia. *Methods*. Institution based cross-sectional study design was conducted among 411 subjects using an Amharic version of the perceived devaluation and discrimination scale. Single population proportion formula was used to calculate sample size. Subjects were selected by systematic sampling techniques. Binary logistic regression and odds ratio with 95% confidence interval were used to identify the association factors of outcome variables. *Results*. A total of 411 subjects participated in the study giving a response rate of 97.4%. The prevalence of perceived stigma was found to be 83.5%. Education status (not able to read and write) (AOR = 2.64, 95% CI: 1.118, 6.227), difficulties of adherence to antipsychotic drug (AOR = 4.49, 95% CI: 2.309, 8.732), and duration of illness less than one year (AOR = 3.48, 95% CI: 2.238, 5.422) were factors associated with perceived stigma. *Conclusion*. Overall, the prevalence of perceived stigma was found to be high. Education status (not able to read and write), difficulties of adherence to antipsychotic medication, and duration of illness were factors associated with perceived stigma. Adherence to antipsychotic medication particularly during the early stage of the illness and strengthening the educational status of the participants were suggested in the clinical care setting.

## 1. Background 

Mental and behavioral disorders are estimated to account for 12% of the global burden of disease [1]. Globally, 450 million people were estimated to be suffering from neuropsychiatric conditions [2]. Of these conditions Schizophrenia is one of the most chronic and disabling illnesses, which affects 24 million people worldwide [2, 3]. In Ethiopia, mental illness is the leading noncommunicable disorder in terms of burden with schizophrenia and depression included in the top ten most burdensome conditions, out-ranking HIV/AIDS [4].

While effective treatment for mental disorders is available, barriers such as stigma against people with mental disorders prevent them from accessing and receiving the help they need to get and stay well [1]. One of the most severely stigmatized mental disorders is schizophrenia, which was selected as the central focus of the World Psychiatric Association's global antistigma programme entitled “Open the Doors” [5, 6].

Stigma is typically a social process, experienced or anticipated and characterized by exclusion, rejection, and blame or devaluation about a person or group [7]. Literature reveals that stigma occurs at three different levels, namely, the macro-, meso-, and microlevel. Institutional stigma refers to the stigma that exists at system (macro) level and was defined as the rules, policies, and procedures of private and public entities in positions of power that restrict the rights and opportunities of people with disabling conditions. Public stigma occurs at the group (meso) level and can be defined as the phenomenon of large social groups endorsing stereotypes about and acting against a stigmatized group [8].

Attitudes toward mental illness vary among individuals, families, ethnicities, cultures, and countries. Cultural and religious teachings often influence beliefs about the origins and nature of the illness and shape attitudes towards the mentally ill [9]. Microlevel stigma is the stigma existing at the individual. Although the term mental illness stigma is often used in a broad sense at microlevel, it can be divided into perceived public stigma/stereotype awareness (participants' beliefs that in general people with mental illness are stigmatized in society), personal stigma/stereotype agreement (participants' personal beliefs about mental illness), and self-stigma or internalized stigma occurs when an individual with mental illness identifies themselves with the stigmatized group and applies corresponding stereotypes and prejudices to the self [10, 11].

Although the reasons for stigmatization are not consistent across communities or cultures, perceived stigma by individuals living with mental illness is associated with different consequences. Different literature reported that perceived stigma affects many domains of the lives of people with schizophrenia such as impacts on self-esteem [12, 13], recovery from the illness, social relationships [14], treatment adherence, willingness to seek help [15], persistent suffering, disability and economic loss [6], and difficulties of accessing housing and employment [16]. These impacts can worsen a client's mental illness and consequently result in involvement in the criminal justice system rather than being treated by the mental health services [17].

Many studies from the Western world have reported on stigmatization of people with mental illnesses and its negative consequences, but few studies have addressed the issue in developing societies. The few studies conducted in Africa reported that stigma regarding mental illness is widespread. A representative survey based on a questionnaire developed for the World Psychiatric Association's Programme to Reduce Stigma and Discrimination because of Schizophrenia was conducted in three Yoruba-speaking communities in Nigeria and the result found predominantly negative views on mental illness across all study subjects [18]. Similar finding was reported from Ghana [19].

In Ethiopia studies revealed that there are widespread beliefs that severe mental illnesses are due to demon possessions, bewitchment by evil spirits, ancestors' spirits, or the evil eyes. As a result, affected individuals and/or their families often seek help from religious and traditional healers rather than health facilities [4]. However, the available studies from Western and some African countries demonstrated high perceived stigma; in Ethiopia there is no previous study that reported on perceived stigma specifically among people with schizophrenia. The only reported study concerning perceived stigma was carried out among family members of individuals with schizophrenia and major affective disorders in rural Ethiopia [20] and internalized stigma among people with schizophrenia [21]. Therefore, in Ethiopia, with its cultural diversity and mix of rural and urban environment, there is a need to understand the stigma perceived by people with schizophrenia. So the aim of this study was to assess the prevalence of perceived stigma and associated factors among individuals with schizophrenia in Ethiopia.

## 2. Methods 

### 2.1. Study Design

Institution based cross-sectional study design was used.

### 2.2. Study Area and Period

The study was conducted at Amanuel Mental Specialized Hospital (AMSH) from March 1 to 30, 2012. AMSH was established in 1930 and is situated in Addis Ababa, the capital city of Ethiopia. It is the only mental health hospital in the country. At the beginning the hospital was established to protect royal families from mentally ill people, not to care for patients with mental illnesses. The hospital has a total of 300 beds of which 277 are for inpatients and 23 are Emergency beds; there is also a large out-patient service, with around 115,000 visiting Outpatients Department each year.

### 2.3. Participants

Participants of this study were individuals with schizophrenia receiving follow-up care at the outpatient department of Amanuel Mental Health Specialized Hospital. Single population proportion formula (with 5% margin of error, 95% confidence level, and 50% proportion) was used to calculate sample size, and it was found to be 422 (including 10% nonresponse rate). The total number of patients who were on follow up care for the last 12 months was taken from patient records and then the average number of patients per day was calculated and participants were selected by systematic random sampling technique. All were consenting individuals with a clinical diagnosis of schizophrenia coming for follow-up, age greater than or equal to 18 years. Individuals with schizophrenia who were unable to speak, hear and have no insight were excluded from the study.

### 2.4. Instrument

The perceived stigma was measured by using the perceived devaluation and discrimination scale (PDD). The PDD is a 12-item tool which measures the extent to which a person believes that most people will devalue or discriminate against someone with a mental illness. PDD was measured on a 4-point Likert scale with possible scores ranging from 1 to 4 agreement scale (1 = strongly disagree, 2 = disagree, 3 = agree, and 4 = strongly agree), so that a higher score indicates a higher level of perceived stigma. This scale has been widely used across the world including Africa [19] and has excellent psychometric properties [22]. Items 1, 2, 3, 4, 8, and 10 were reverse-scored.

The questionnaires were translated into Amharic language by an Amharic speaking linguist. The back-translations were performed by a psychiatrist into English and then a consensus version was developed in a group discussion by involving another psychiatrist and one Amharic speaking linguist. This was compared with the original version and confirmed to be satisfactory for use. In this study, PDD had an internal consistence of Cronbach's alpha for the total score was 0.79. The questionnaires were tested on 21 patients to make it easier for the participants to understand and complete.

The prevalence of high perceived stigma was defined as an item mean score of 2.5 or higher on mean aggregated scale score (this criterion represented the “midpoint” on the 1–4-item scale) on PDD scales. Then perceived stigma scores were dichotomized as those participants scoring greater than or equal to the mean score of 2.5 on PDD scales as having “high perceived stigma” and those scoring below the mean score as having “low perceived stigma.”

Furthermore, we asked two questions on antipsychotic medication adherence and difficulties of patients' adherence to their clinic appointments (follow-up). The antipsychotic medication adherence question asked about history of nonadherence with antipsychotic medications and whether the nonadherence behavior was linked to stigma with yes/no response. Specifically, we asked, “have you ever discontinued your antipsychotic medication because of fear of perceived stigma associated with your mental illness?” “Have you ever discontinued your clinic appointments (follow-up) because of fear of perceived stigma associated with your mental illness?”

### 2.5. Data Collection and Analysis

Data were collected by face-to-face interview using a semistructured questionnaire with the Amharic version of the sociodemographic, clinical factors, and perceived devaluation and discrimination questionnaires.

Data were coded and entered into EPI info version 3.5.3 statistical software and then exported to SPSS windows version 16 program for analysis. Descriptive statistics (frequencies, tables, percentages, means, and standard deviation) were used for the sociodemographic and clinical variables including individual's response items to PDD. Binary logistic regression and odds ratio with 95% confidence interval were used to identify the associated factors of outcome variable. A significance level of 0.05 was taken as cut-off value for all statistical significance tests.

### 2.6. Ethical Consideration

The study proposal was initially approved by the ethical review board of The University of Gondar and Amanuel Mental Specialized Hospital. A formal letter of permission was obtained from the hospital and submitted to the respective Outpatient Department. The information about the study was given to the participants. Verbal then written informed consent was sought for each participant who agreed to participate in the study and fulfilled the inclusions criteria. Only anonymous data were collected in private rooms.

## 3. Results

A total of 411 participants participated in this study with a 97.4% response rate. One of the participants failed to complete the interview because of the illness and nine questions were not fulfilled properly.

### 3.1. Sociodemographic Characteristics

The majority of the participants were males 302 (73.5%). The mean ages of the participants were 33.24 ± 9.73 years. Two hundred and eighty-nine (70.3%) of the participants were primary educated, 240 (58.4%) were Orthodox, and 286 (69.6%) were single in marital status. One hundred and six (35.5%) of the participants were Amhara in Ethnicity. Out of 411 participants, 257 (62.5%) were unemployed, 322 (78.3%) were living in urban areas, and 360 (87.6%) were living with their family (Table 1).

### 3.2. Clinical Characteristics

Regarding the clinical characteristics of participants, 117 (28.5%) were treated for 2–5 years, 287 (69.8%) had difficulties of attending clinic (follow-up), and 64 (15.6%) had difficulties of adherence to antipsychotic medication (Table 2).

### 3.3. Prevalence of Perceived Stigma

Overall, the prevalence of perceived stigma was found to be 83.5%. Regarding the proportion of perceived stigma toward each item, the majority (362) (88.07%) of the participants agreed with the item “most people think that a person who has been hospitalized for mental illness is dangerous and unpredictable” (Table 3).

### 3.4. Factors Associated with Perceived Stigma

From the bivariate analysis, educational statuses, employment, residence, duration of illness, and difficulties of adherence to antipsychotic medication were factors associated with perceived stigma in bivariate analyses and entered into multivariate analysis.

On the other hand, age, sex, ethnicity, marital status, religion, difficulties of adherence to clinic (follow-up), and living arrangement were not associated and were excluded from further analysis.

From the multivariate analysis, education status (not able to read and write) (AOR = 2.64, 95% CI: 1.118, 6.227), difficulties of adherence to antipsychotic medication (AOR = 4.49, 95% CI: 2.309, 8.732), and duration of illness ≤ 1 year (AOR = 3.48, 95% CI: 2.238, 5.422) were factors statistically significant with perceived stigma (Table 4).

## 4. Discussion

The aim of this study was to assess the prevalence of perceived stigma and associated factors among people with schizophrenia at Amanuel Mental Specialized Hospital. This study shows that overall the prevalence of perceived stigma was found to be 83.5%. This study was higher when compared with the cross-sectional study carried out across 14 European countries using the same tool. That study reported 69.4% [22] and a systematic review of studies including 54 published studies from 1994 to 2011 also reported on average 64.5% of participants' perceived stigma [23].

This variation may be due to shared social perception toward mental illness. A previous study relatives of schizophrenia and affective disorder revealed that 75% of respondents' had perceived stigma in Ethiopia and since the study subjects were drawn from these societies, they may share the society's perception about mental illness stigma [20]. This relationship indicates or supports the evidence of relation between public stigma toward mental illness and perceived stigma. This variation could be also due to the differences in sample size, the population surveyed in the setting, method of data collection, and lack of antistigma interventions program in the study area.

Even if the ways in which the respondents' experience of perceived stigma toward each item was different from the study done in Ghana using the same tool because of differences in the culture, setting, and other factors, there are also a number of similarities reported. For example, 85.7% of Ghanaian participants agreed with the item “opinions of a mentally ill person were taken less seriously” compared to 81% in this study and 77.2% of the participants agreed with the item “most employers would pass over an application of a former mental patient in favour of another applicant” compared to 76.1% in this study [19].

Regarding the associated factors, those patients who cannot read and write were more than two times (AOR = 2.64, 95% CI: 1.118, 6.227) more likely to develop perceived stigma than those patients who had educational status of secondary level and above. These results were consistent with the previous study [12, 24–26]. This is due to the fact that those patients who cannot read and write may have poor coping strategies to perceived stigma.

From clinical variables those patients who had difficulties of adherence to their antipsychotic medication were more than four times (AOR = 4.49, 95% CI: 2.309, 8.732) more likely to experience perceived stigma than those patients who adhere to their medication. These results were consistent with the meta-analysis carried out among one hundred and twenty-seven studies in Europe [15, 27, 28]. This may be due to fear of stigma and rejection which could prolong the duration of help seeking and reduce the likelihood of adherence to treatment. This may in turn result in an increased risk of socially unacceptable violent behavior which creates stigma.

Those patients who had less than or equal to one year duration of illness had more than three times (AOR = 3.48, 95% CI: 2.238, 5.422) perceived stigma than those patients who had greater than one year duration of their illness. These results were consistent with the previous studies in India and Europe [22, 23, 29]. This may be due to the presence of socially unacceptable behavior seen predominantly during the acute phase of the illness. Behaviours such as hallucinations, delusions, and suspicion were found to be very distressing and associated with the creation of stigma. Another possible reason may be that since perceived stigma was the result of perception of public stigma, through time, the patient may develop stigma resistance through experience.

### 4.1. Strength of the Study

This study is the first of its kind in Ethiopia to show perceived stigma among people with schizophrenia using a standard tool.

### 4.2. Limitations of the Study

The lack of published literature in Ethiopia limits the comparison (discussion) of the findings. Recall and response biases might have occurred when completing the questionnaire. In addition, some of the independent variables were assessed with single questions, for example, treatment adherence and difficulties of follow-up adherence to their clinic appointments.

## 5. Conclusion 

Overall, the prevalence of perceived stigma was found to be high among the study participants. Lower educational status, difficulties of adherence to antipsychotic medication, and less than or equal to one year duration of illness were factors associated with perceived stigma. These findings add important evidence to the existing scant study in Sub-Saharan Africa and other developing countries on the psychosocial aspect of individual with schizophrenia as perceived stigma is an important area to improve patients' adherence to antipsychotic medication. Therefore stigma focused intervention such as adherence to antipsychotic medication particularly during the early stage of the illness and strengthening the educational status of the participants were suggested in the clinical care setting. Additional researches with qualitative and quantitative study methods are also suggested, in order to explore the relation of sociodemographic and perceived stigma.

## Figures and Tables

**Table 1:** Sociodemographic characteristics of participants (*n* = 411) at Amanuel Mental Specialized Hospital, 2012.

Characteristics	Number	Percent
Sex		
Male	302	73.5
Female	109	26.5
Age		
18–24	84	20.4
25–34	162	39.4
35–44	114	27.7
≥44	51	12.4
Educational status		
Cannot read and write	40	9.5
Primary	289	70.3
Secondary and above	52	20
Religion		
Muslim	91	22.1
Orthodox	240	58.4
Protestant	67	16.3
Catholic	13	3.2
Marital status		
Married	72	17.5
Single	286	69.6
Divorced/widowed	53	12.8
Ethnicity		
Oromo	132	32.1
Amhara	146	35.6
Gurage	98	23.8
Tigre	35	8.5
Employment		
Unemployed	257	62.5
Employed	154	37.5
Residence		
Rural	89	21.7
Urban	322	78.3
Living arrangement		
Family	360	87.6
Alone	51	12.4

**Table 2:** Distribution of participants (*n* = 411) by clinical characteristics at Amanuel Mental Specialized Hospital, 2012.

Characteristics	Frequency	Percent
Duration of the treatment (in years)		
≤1	101	24.5
2–5	117	28.5
6–10	90	21.9
≥11	103	25.1
Duration of illness (in years)		
≤1	204	49.6
2–5	78	19
6–10	45	10.9
≥11	84	20.5
Follow-up adherence to clinic appointment		
Yes	287	69.8
No	124	30.2
Difficulty of adherence to antipsychotic drug		
Yes	64	15.6
No	347	84.4

**Table 3:** Proportion of perceived stigma response of participants (*n* = 411) to each item at Amanuel Mental Specialized Hospital, 2012.

	Items	Strongly disagree	Disagree	agree	Strongly agree
1	Most people would be close friends with a person who once had severe mental illness.	22	267	116	6
2	Most people believe that a person who has severe mental illness is just as intelligent as anyone else.	23	280	106	2
3	Most people believe that a person who has been treated for severe mental illness is just as trustworthy as anyone else.	12	246	151	2
4	Most people would accept a person who has had severe mental illness as a teacher in a school.	24	297	81	9
5	Most people believe that receiving treatment for severe mental illness is a sign of personal failure.	3	101	291	16
6	Most people will not hire a person who has been hospitalized for severe mental illness to take care of their children, even if he or she had been well for some time.	3	174	214	20
7	Most people think less of a person who has been treated for severe mental illness.	16	62	288	45
8	Most employers will hire a qualified person even if he or she has been treated for severe mental illness.	18	161	227	5
9	Most employers would prefer to hire someone who does not have a history of severe mental illness.	5	93	298	15
10	Most people I know would treat a person who has been treated for severe mental illness the same way they treat everyone else.	9	157	239	6
11	Most young women would be reluctant to date a man who has been treated for severe mental illness.	37	358	12	4
12	Most people think that a person who has been hospitalized for severe mental illness is dangerous and unpredictable.	3	46	346	16

**Table 4:** Factors associated with perceived stigma (bivariate and multivariate) analysis, at Amanuel Mental Specialized Hospital, 2012.

Explanatory variables	Perceived stigma		COR (95% CI)	AOR (95% CI)
	High	Low		
Education				
Not able to read and write	27	13	2.93 (1.325, 6.488)	**2.64 (1.118, 6.227)**
Primary	143	146	1.38 (0.842, 2.271)	
Secondary and above	34	48	1	1
Employment				
Unemployed	135	122	1.36 (1.213, 4.036)	**∗**
Employed	69	85	1	
Residence				
Rural	51	38	1.48 (1.323, 5.38)	∗
Urban	153	169	1	1
Difficulty of adherence to antipsychotic medication				
Yes	43	21	2.37 (1.347, 4.153)	**4.49 (2.309, 8.732)**
No	161	186	1	1
Duration of illness				
>1 year	81	126	1	1
≤1 year	131	73	2.79 (1.872, 4.164)	**3.48 (2.238, 5.422)**

Note: *not statistically significant using forward stepwise regression methods. Hosmer and Lemeshow test was 0.191.
